# Correction: The AGC protein kinase UNICORN controls planar growth by attenuating PDK1 in *Arabidopsis thaliana*

**DOI:** 10.1371/journal.pgen.1008170

**Published:** 2019-05-14

**Authors:** Sebastian Scholz, Janys Pleßmann, Balaji Enugutti, Regina Hüttl, Katrin Wassmer, Kay Schneitz

There are errors in the labelling of panels in Fig 5.

Specifically, in panel A, the domain label second from the top should read ‘UCNG165S’ rather than ‘UCN165S’. In panels B and C, labels for lanes 5 and 6 should be swapped, with lane 5 correctly labelled as ‘GST:UCNKD’ and lane 6 as ‘GST:UCNdeltaPIF’. In panels F and G, labels for lanes 6 and 7 should be swapped, with lane 6 correctly labelled as ‘GST:UCNKD’ and lane 7 as ‘GST:UCNdeltaPIF’. In panels B and F, the horizontal bar above +MBP:PDK1.1 and +MBP:PDK1.1KD is missing. In panel F, the rightmost lane should be labelled as ‘MBP:PDK1.1KD’. In panel G, the rightmost lane should be labelled as ‘MBP:PDK1.2KD’. A corrected version is provided below.

Additionally, an image is missing from Fig 8D. A corrected version is provided below.

**Fig 5:**
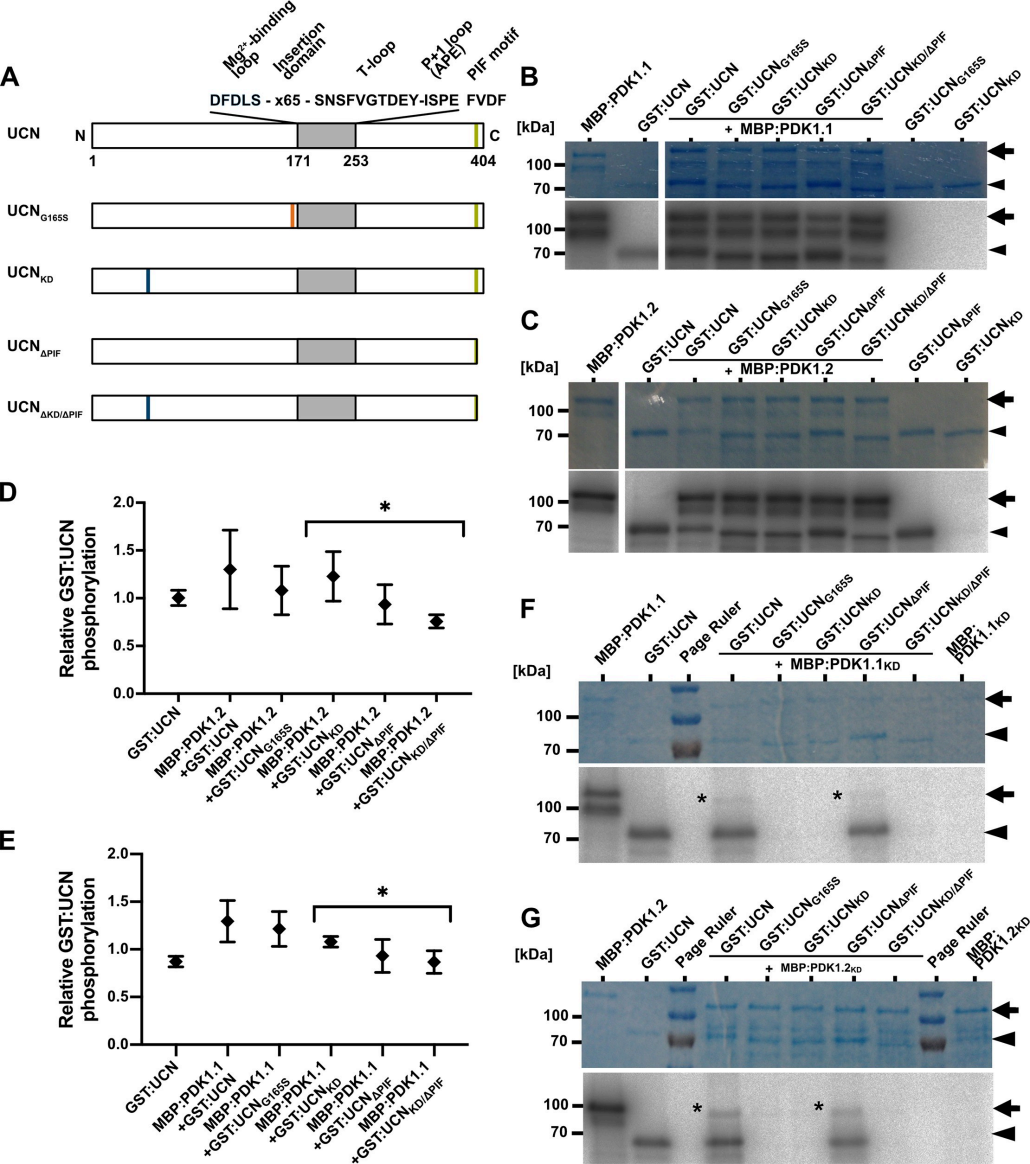
In vitro kinase assays with PDK1 in combination with different UCN variants. (A) Cartoon outlining the domain structure of UCN and marking the alterations in the different mutant variants. The plant AGC protein kinase specific insertion domain in the activation segment is also indicated. (B,C) and (F,G) Shown are coomassie brilliant blue (CBB)-stained gels (upper panel) and autoradiograms (lower panel) for MBP:PDK1.1, MBP:PDK1.2, MBP:PDK1.1_KD_, and MBP:PDK1.2_KD_, respectively, in combination with different GST:UCN versions. Arrows indicate the various MBP:PDK1 forms, arrowheads mark the different GST:UCN variants. (B) Kinase assays involving MBP:PDK1.1. Note the presence of corresponding labelled bands for all variants of GST:UCN. (C) Kinase assays involving MBP:PDK1.2. Note the presence of corresponding labelled bands for all variants of GST:UCN. (F) Kinase assays involving MBP:PDK1.1_KD_. Asterisk indicates MBP:PDK1.1_KD_ band weakly phosphorylated by GST:UCN or GST:UCN_ΔPIF_. (G) Kinase assays involving MBP:PDK1.2_KD_. Asterisk indicates MBP:PDK1.2_KD_ band weakly phosphorylated by GST:UCN or GST:UCN_ΔPIF_. (D,E) Intensity-based quantification of the bands indicated by the arrowheads and arrows in (B,C and F,G) (autoradiogram signal relative to the corresponding CBB gel band intensity) using ImageJ/Fiji [80,81]. The results of three independent experiments (involving protein induction, purification and kinase assays) are shown. Please note statistically significant differences of phosphorylation intensity in a PIF motif-dependent manner (asterisks): (D) p = 0.031; (E) p = 0.039.

**Fig 8:**
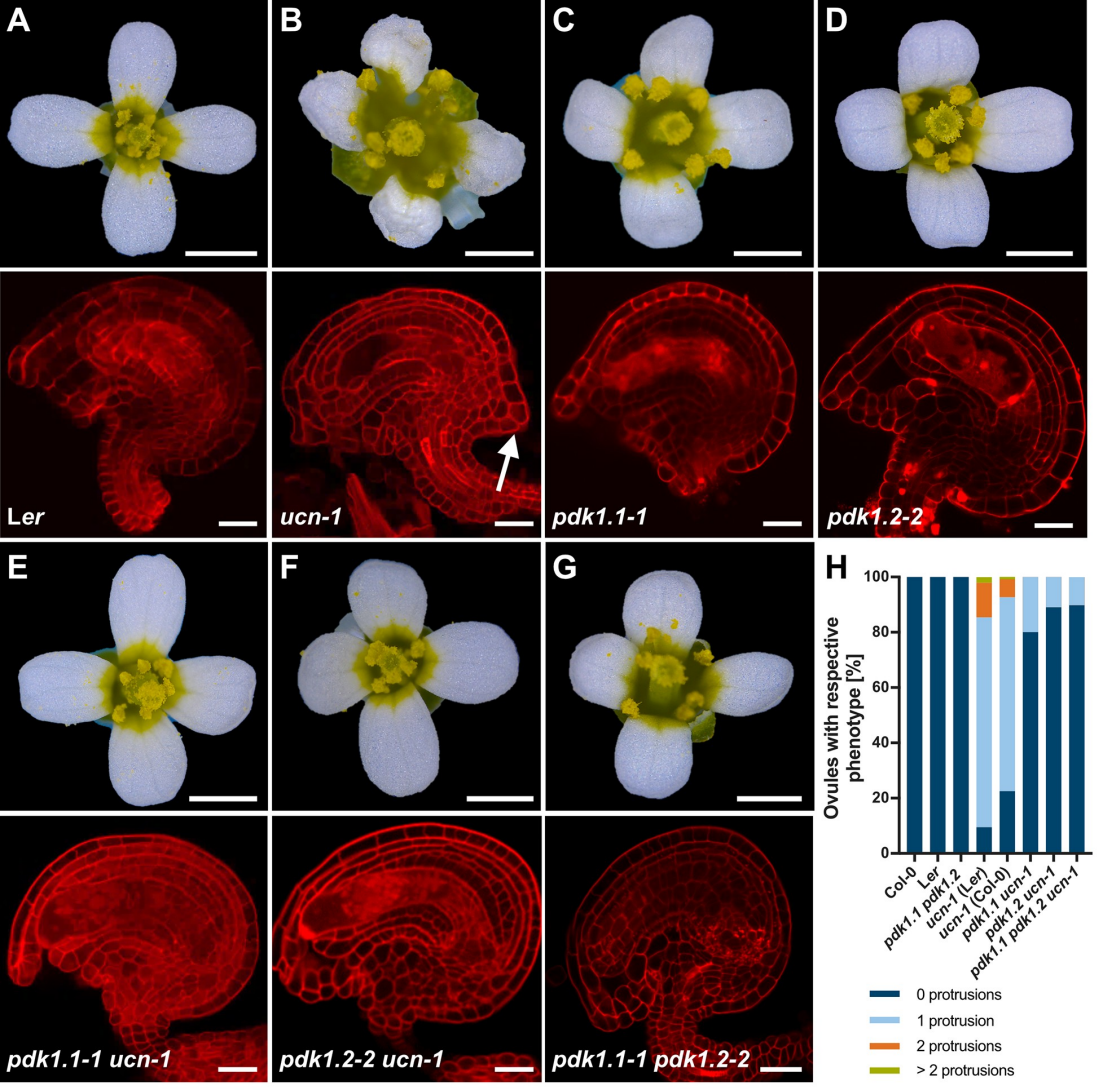
Analysis of pdk1, ucn-1 and pdk1 ucn-1 floral and ovule phenotypes. (A-F) Upper panel: Stage 13 flowers are shown (stages according to [82]). Lower panel: Confocal micrographs show about mid-optical sections through late stage 3 or stage 4 mPS-PI-stained ovules. Genotypes are indicated. (B) Upper panel: Note aberrant petal shape. Lower panel: arrow indicates ectopic protrusion. (C-G) Apparently normal phenotypes (compare with (A)). (H) Percentage of respective ovule phenotypes of L*er*, *ucn-1*, Col-0, *ucn-1* outcrossed to L*er* and Col-0 (F3 plants homozygous for *ucn-1*), respectively, and homozygous double mutants (*pdk1*.*1 ucn-1* and *pdk1*.*2 ucn-1*) and homozygous triple mutants (*pdk1*.*1 pdk1*.*2 ucn-1*). Knockouts of *pdk1*.*1*, *pdk1*.*2* or both restore the *ucn-1* phenotype to 73% and 90% of the WT level, respectively (*ucn-1 pdk1*.*1–2*: 73%; *ucn-1 pdk1*.*1–1*: 80%; *ucn-1 pdk1*.*2–3*: 86%; *ucn-1 pdk1*.*2–2*: 90%). Sample sizes are given in Table 1. Scale bars: (A-F) Upper panels, 1mm; lower panels, 20 μm.
