# Supplementary material for: The AGC protein kinase UNICORN controls planar growth by attenuating PDK1 in Arabidopsis thaliana
Source: PLoS Genet. 2019 Feb 11;15(2):e1007927. doi: 10.1371/journal.pgen.1007927 (PMC6386418; doi:10.1371/journal.pgen.1007927)
Supplement: S1 Table — (DOCX) [file pgen.1007927.s001.docx]

**S1 Table. Primers used in this study.**

| **Name** | **5'-sequence-3'** | **Purpose** |
| --- | --- | --- |
| PDK1.1_fw EcoRI_new | GGCCGAATTCATGTTGGCAATGGAGAAAGAA | Cloning PDK1.1 CDS into pMal c2x forward |
| PDK1.1_rev BamHI_new | GGCCGGATCCTCAGCGGTTCTGAAGAGTCTC | Cloning PDK1.1 CDS into pMal c2x reverse |
| PDK1.2 fw_BamHI_new | GGCCGGATCCATGTTGACAATGGACAAGGAA | Cloning PDK1.2 CDS into pMal c2x forward |
| PDK1.2_rev_PstI_new | GGATCTGCAGTCAACGGTTTTGAAGAGTTTC | Cloning PDK1.2 CDS into pMal c2x reverse |
| PDK1.1_fw_BamHI_BiFC | CCCCGGATCCATGTTGGCAATGGAGAAAGAATTT | Cloning PDK1.1 CDS into pSPYCE/pSPYNE forward |
| PDK1.1_rev_XmaI_BiFC | AAAACCCGGGGCGGTTCTGAAGAGTCTCGAT | Cloning PDK1.1 CDS into pSPYCE/pSPYNE reverse |
| PDK1.2_fw_BamHI_BiFC | CCCCGGATCCATGTTGACAATGGACAAGGAA | Cloning PDK1.2 CDS into pSPYCE/pSPYNE forward |
| PDK1.2_rev_KpnI_BiFC | CCCCGGTACCACGGTTTTGAAGAGTTTCGAT | Cloning PDK1.2 CDS into pSPYCE/pSPYNE reverse |
| UCN_fw_XbaI_BiFC | CCCCTCTAGAATGGAGACAAGACCATCATCATCATC | Cloning UCN into pSPYCE/pSPYNE forward |
| UCN_dPIF_rev_XmaI_BiFC | AAAACCCGGGCGGATTGTTTTCAGAACACTCGTGC | Cloning UCN_dPIF into pSPYCE/pSPYNE reverse |
| UCN_rev_XmaI_BiFC | AAAACCCGGGGAAATCAACAAACGGATTGTTTTCAGA | Cloning of UCN into pSPYCE/pSPYNE reverse |
| SALK_LBb1.3 | ATTTTGCCGATTTCGGAAC | Genotyping SALK lines |
| pdk1.1_SALK_053385_LP | TGGAAGTTTGGTTGATCGAAG | Genotyping pdk1.1-1 SALK_053385 |
| pdk1.1_SALK_053385_RP | ACATTAGCACCGTTGGATGAG | Genotyping pdk1.1-1 SALK_053385 |
| pdk1.1_SALK_113251_LP | TGGTAAGATGCATCAAAAGCC | Genotyping pdk1.1-2 SALK_113251 |
| pdk1.1_SALK_113251_RP | TTACCACGGTTTTGTGAAAGG | Genotyping pdk1.1-2 SALK_113251 |
| SAIL_LB2 | GCTTCCTATTATATCTTCCCAAATTACCAATACA | Genotyping SAIL lines |
| pdk1.2_SAIL_62_G04_LP | CTTACCATGATTTCGAGCTCG | Genotyping pdk1.2-2 SAIL_62_G04 |
| pdk1.2_SAIL_62_G04_RP | TTCAGGAGGAACATATGCAGC | Genotyping pdk1.2-2 SAIL_62_G04 |
| pdk1.2_SAIL_450_B01_LP | CTTGATCAACTCGAACATCCC | Genotyping pdk1.2-3 SAIL_450_B01 |
| pdk1.2_SAIL_450_B01_RP | AACCTTCTGATCCAGCTCCTG | Genotyping pdk1.2-3 SAIL_450_B01 |
| pUBQ10_HindIII_fw | AACCAAGCTTAGCTGCGACGAGTCAGTAATAAACG | Replacing p35S in pMDC43/83 forward |
| pUBQ10_KpnI_rev | TTCCGGTACCAGATCATGTTAATCAGAAAAACTCAG | Replacing p35S in pMDC43 reverse |
| pUBQ10_SpeI_rev | CCCCACTAGTTGTTAATCAGAAAAACTCAG | Replacing p35S in pMDC83 reverse |
| p16_HindIII_fw | AACCAAGCTTAGCTGTGGAACCATCTTTTGGGTTCC | Replacing p35S in pMDC43/83 forward |
| p16_KpnI_rev | TTCCGGTACCAGATCGACCACGCCGTCGTAGATGAG | Replacing p35S in pMDC43 reverse |
| p16_SpeI_rev | CCCCACTAGTCCACGCCGTCGTAGATGAG | Replacing p35S in pMDC83 reverse |
| pPDK1.2_fw_attB1 | GGGGACAAGTTTGTACAAAAAAGCAGGCTATGGGTTTTCCACCTTGAGGC | Cloning pPDK1.2::gPDK1.2 into pMDC43 forward |
| PDK1.2_rev_attB2 | GGGGACCACTTTGTACAAGAAAGCTGGGTTACGGTTTTGAAGAGTTTCG | Cloning pPDK1.2::gPDK1.2 into pMDC43 reverse |
| PDK1.1sense_CDS_F | TAATACGACTCACTATAGGGATGTTGGCAATGGAGAAAG | PDK1.1 in situ sense probe forward |
| PDK1.1sense_CDS_R | TCAGCGGTTCTGAAGAGTC | PDK1.1 in situ sense probe reverse |
| PDK1.1as_CDS_F | ATGTTGGCAATGGAGAAAG | PDK1.1 in situ antisense probe forward |
| PDK1.1as_CDS_R | TAATACGACTCACTATAGGGTCAGCGGTTCTGAAGAGTC | PDK1.1 in situ antisense probe reverse |
| PDK1.2sense_CDS_F | TAATACGACTCACTATAGGGATGTTGACAATGGACAAGG | PDK1.2 in situ sense probe forward |
| PDK1.2sense_CDS_R | TCAACGGTTTTGAAGAGTT | PDK1.2 in situ sense probe reverse |
| PDK1.2as_CDS_F | ATGTTGACAATGGACAAGG | PDK1.2 in situ antisense probe forward |
| PDK1.2as_CDS_R | TAATACGACTCACTATAGGGTCAACGGTTTTGAAGAGTT | PDK1.2 in situ antisense probe reverse |
| At4g33380(qRT)_F | TGAAGGAGAGGAAGAGCCTGAGGAA | Reference gene 1 forward qRT-PCR |
| At4g33380(qRT)_R | CCCCATCTCACTGCAGCACCAC | Reference gene 1 reverse qRT-PCR |
| At2g28390(qRT)_F | AGATTGCAGGGTACGCCTTGAGG | Reference gene 2 forward qRT-PCR |
| At2g28390(qRT)_R | ACACGCATTCCACCTTCCGCG | Reference gene 2 reverse qRT-PCR |
| At5g46630(qRT)_F | CCAAATGGAATTTCAGGTGCCAATG | Reference gene 3 forward qRT-PCR |
| At5g46630(qRT)_R | CAATGCGTACCTTGAGAAAACGAAC | Reference gene 3 reverse qRT-PCR |
| PDK1.2_(qRT)_fw | TCGCCTTTAAGGCTCCTCAGG | PDK1.2 forward qRT-PCR |
| PDK1.2_(qRT)_rev | CCATGATCTTCAAGGCATACACAG | PDK1.2 reverse qRT-PCR |
| PDK1.1(qRT)_fw2 | AACGGTGCTAATGTTTCTAGAAGC | PDK1.1 forward qRT-PCR |
| PDK1.1(qRT)_rev | CAGTTTCCTTCTTCTTTGCCCTAAC | PDK1.1 reverse qRT-PCR |
