# Supplementary material for: The AGC protein kinase UNICORN controls planar growth by attenuating PDK1 in Arabidopsis thaliana
Source: PLoS Genet. 2019 Feb 11;15(2):e1007927. doi: 10.1371/journal.pgen.1007927 (PMC6386418; doi:10.1371/journal.pgen.1007927)
Supplement: S1 Fig — A previous study had suggested a third PDK1-like gene for Arabidopsis thaliana (At2g20050) [83]. However, protein sequence comparison failed to confirm any similarity of At2g20050 with PDK1. The amino acid alignment was performed using ClustalW algorithm and BLOSUM62 matrix in Geneious 11.1.5 software (https://www.geneious.com). Amino acids are highlighted in color. Lines represent gaps. Please note that At2g20050 consists of 1,094 amino acids (PDK1.1 491, PDK1.2486). (DOCX) [file pgen.1007927.s002.docx]

**S1 Fig.** **Protein alignment of PDK1.1, PDK1.2, and At2g20050.**

A previous study had suggested a third *PDK1*-like gene for *Arabidopsis thaliana* (At2g20050) [83]. However, protein sequence comparison failed to confirm any similarity of At2g20050 with *PDK1*. The amino acid alignment was performed using ClustalW algorithm and BLOSUM62 matrix in Geneious 11.1.5 software (https://www.geneious.com). Amino acids are highlighted in color. Lines represent gaps. Please note that At2g20050 consists of 1,094 amino acids (PDK1.1 491, PDK1.2486).
