# Supplementary material for: The AGC protein kinase UNICORN controls planar growth by attenuating PDK1 in Arabidopsis thaliana
Source: PLoS Genet. 2019 Feb 11;15(2):e1007927. doi: 10.1371/journal.pgen.1007927 (PMC6386418; doi:10.1371/journal.pgen.1007927)
Supplement: S2 Fig — (a) pdk1.1–1 pdk1.2–2 (b) pdk1.1–2 pdk1.2–3. (c) Col-0. (d) pdk1.1–1 pdk1.2–2 p16::gPDK1.1:EGFP. (e) pdk1.1–1 pdk1.2–2 p16::gPDK1.2:EGFP. (f) pdk1.1–2 pdk1.2–3 pUBQ10::EGFP:gPDK1.1. (g) pdk1.1–2 pdk1.2–3 pUBQ10::EGFP:gPDK1.2. (h) pdk1.1–1 pdk1.2–2 pPDK1.2::gPDK1.2:EGFP. All constructs restore plant height. Dashed line indicates maximal plant height of the double mutants. Scale bar: 10 cm. (DOCX) [file pgen.1007927.s003.docx]

**S2 Fig. Restoration of *pdk1.1 pdk1.2* phenotype by transgenes expressing translational fusions of PDK1 to EGFP.**

(a) *pdk1.1-1 pdk1.2-2* (b) *pdk1.1-2 pdk1.2-3*. (c) Col-0. (d) *pdk1.1-1 pdk1.2-2 p16::gPDK1.1:EGFP*. (e) *pdk1.1-1 pdk1.2-2 p16::gPDK1.2:EGFP*. (f) *pdk1.1-2 pdk1.2-3 pUBQ10::EGFP:gPDK1.1*. (g) *pdk1.1-2 pdk1.2-3 pUBQ10::EGFP:gPDK1.2*. (h) *pdk1.1-1 pdk1.2-2 pPDK1.2::gPDK1.2:EGFP*. All constructs restore plant height. Dashed line indicates maximal plant height of the double mutants. Scale bar: 10 cm.
