# Supplementary material for: The AGC protein kinase UNICORN controls planar growth by attenuating PDK1 in Arabidopsis thaliana
Source: PLoS Genet. 2019 Feb 11;15(2):e1007927. doi: 10.1371/journal.pgen.1007927 (PMC6386418; doi:10.1371/journal.pgen.1007927)
Supplement: S3 Fig — Confocal micrographs are shown. (A) pUBQ10::PDK1.2:EGFP. (B,C) pUBQ10::PDK1.1:EGFP. (A) Filament, (AI) higher magnification of the region in (A), (B) ovule, (C) transition zone of a root, and (CI) higher magnification of the region in (C). Arrows in (AI) indicate the absence of GFP signal at the cell walls, magenta arrows in (CI) indicate most likely endoplasmic reticulum (ER), arrowheads indicate plasma membranes and asterisks indicate nuclei. Scale bars: 20 μm. (DOCX) [file pgen.1007927.s004.docx]

**S3 Fig. Subcellular localization of pUBQ::PDK1:EGFP reporter signals.**

Confocal micrographs are shown. (A) pUBQ10::PDK1.2:EGFP. (B,C) pUBQ10::PDK1.1:EGFP. (A) Filament, (A^I^) higher magnification of the region in (A), (B) ovule, (C) transition zone of a root, and (C^I^) higher magnification of the region in (C). Arrows in (A^I^) indicate the absence of GFP signal at the cell walls, magenta arrows in (C^I^) indicate most likely endoplasmic reticulum (ER), arrowheads indicate plasma membranes and asterisks indicate nuclei. Scale bars: 20 μm.
