# Supplementary material for: The AGC protein kinase UNICORN controls planar growth by attenuating PDK1 in Arabidopsis thaliana
Source: PLoS Genet. 2019 Feb 11;15(2):e1007927. doi: 10.1371/journal.pgen.1007927 (PMC6386418; doi:10.1371/journal.pgen.1007927)
Supplement: S4 Fig — Arabidopsis mesophyll protoplasts were used. The N-terminal part of YFP was fused to PDK1.1, PDK1.2, or UCN, respectively, and the C-terminal part of YFP was fused to different mutant versions of UCN. The variants are indicated. (A-C) Note absence of signal (n > 1500). (D) Signal is localized to the nucleus (126/425 scored protoplasts). Also compare to Fig 4 in [9]. (E,F) No signal is observed (n > 1500). N = 3. Scale bars: 5 μm. (DOCX) [file pgen.1007927.s005.docx]

**S4 Fig. BiFC assays of PDK1 with mutant versions of UCN.** Arabidopsis mesophyll protoplasts were used. The N-terminal part of YFP was fused to PDK1.1, PDK1.2, or UCN, respectively, and the C-terminal part of YFP was fused to different mutant versions of UCN. The variants are indicated. (A-C) Note absence of signal (n > 1500). (D) Signal is localized to the nucleus (126/425 scored protoplasts). Also compare to Fig. 4 in [2]. (E,F) No signal is observed (n > 1500). N = 3. Scale bars: 5 μm.
