# Supplementary material for: The AGC protein kinase UNICORN controls planar growth by attenuating PDK1 in Arabidopsis thaliana
Source: PLoS Genet. 2019 Feb 11;15(2):e1007927. doi: 10.1371/journal.pgen.1007927 (PMC6386418; doi:10.1371/journal.pgen.1007927)
Supplement: S5 Fig — (A) Kinase assays. Upper panel depicts autoradiogram. Lower panel shows corresponding coomassie brilliant blue (CBB)-stained gel. (upper panel) and autoradiograms (upper panel). Assays were performed for MBP:PDK1.1, MBP:PDK1.2, and GST:UCN, respectively, in combination with Trx:ATS. There is weak Trx:ATS phosphorylation signal in combination with MBP:PDK1 in comparison to GST:UCN/Trx:ATS (note the differences in the amount of Trx:ATS involved in the reactions). (B) BiFC assays of PDK1 and ATS. Note absence of signal. N = 3. Abbreviation: DIC, differential interference contrast. Scale bars: 5 μm. (DOCX) [file pgen.1007927.s006.docx]

**S5 Fig. PDK1 phosphorylates ATS in vitro.** (A) Kinase assays. Upper panel depicts autoradiogram. Lower panel shows corresponding coomassie brilliant blue (CBB)-stained gel. (upper panel) and autoradiograms (upper panel). Assays were performed for MBP:PDK1.1, MBP:PDK1.2, and GST:UCN, respectively, in combination with Trx:ATS. There is weak Trx:ATS phosphorylation signal in combination with MBP:PDK1 in comparison to GST:UCN/Trx:ATS (note the differences in the amount of Trx:ATS involved in the reactions). (B) BiFC assays of PDK1 and ATS. Note absence of signal. N = 3. Abbreviation: DIC, differential interference contrast. Scale bars: 5 μm.
